# Supplementary figures and images for: Impact of maternal compensation on developmental phenotypes in a zebrafish model of severe congenital muscular dystrophy
Source: PLoS Genet. 2026 Jan 14;22(1):e1011987. doi: 10.1371/journal.pgen.1011987 (PMC12803454; doi:10.1371/journal.pgen.1011987)

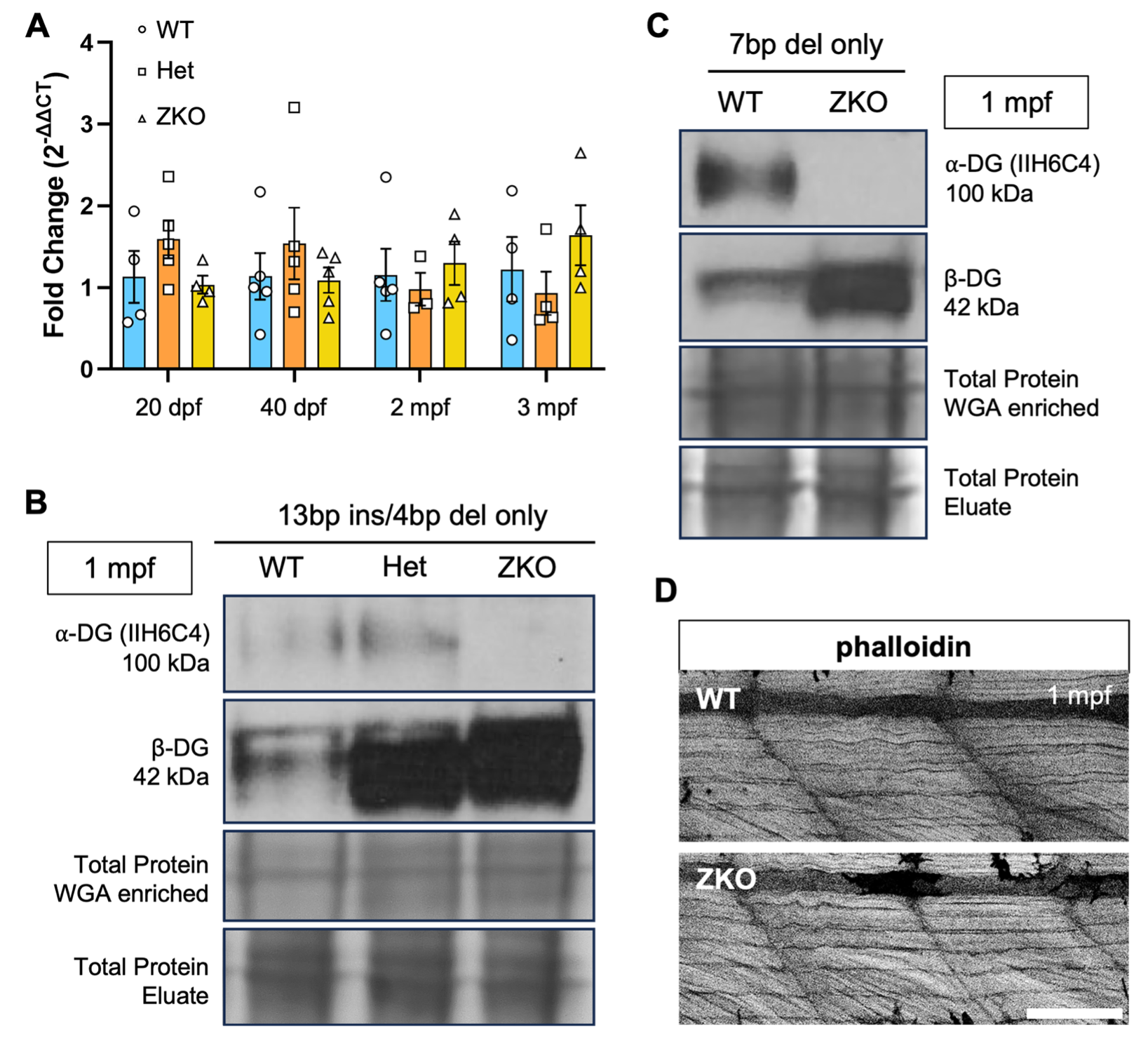

Supplement: S1 Fig — A: Quantitative real-time PCR analysis of pomgnt2 gene expression showing no significant differences in WTs, Hets, and ZKOs. B, C: Western blot analysis of glycoprotein enriched lysate showing that ZKOs with only the exon 1 (B) or exon 2 (C) mutations also show complete loss of α-DG glycosylation. D: Staining of muscle at 1 mpf with fluorescently conjugated phalloidin showing normal muscle fiber integrity in ZKOs (Scale Bar: 100 µm). (TIF) [file pgen.1011987.s001.tif]

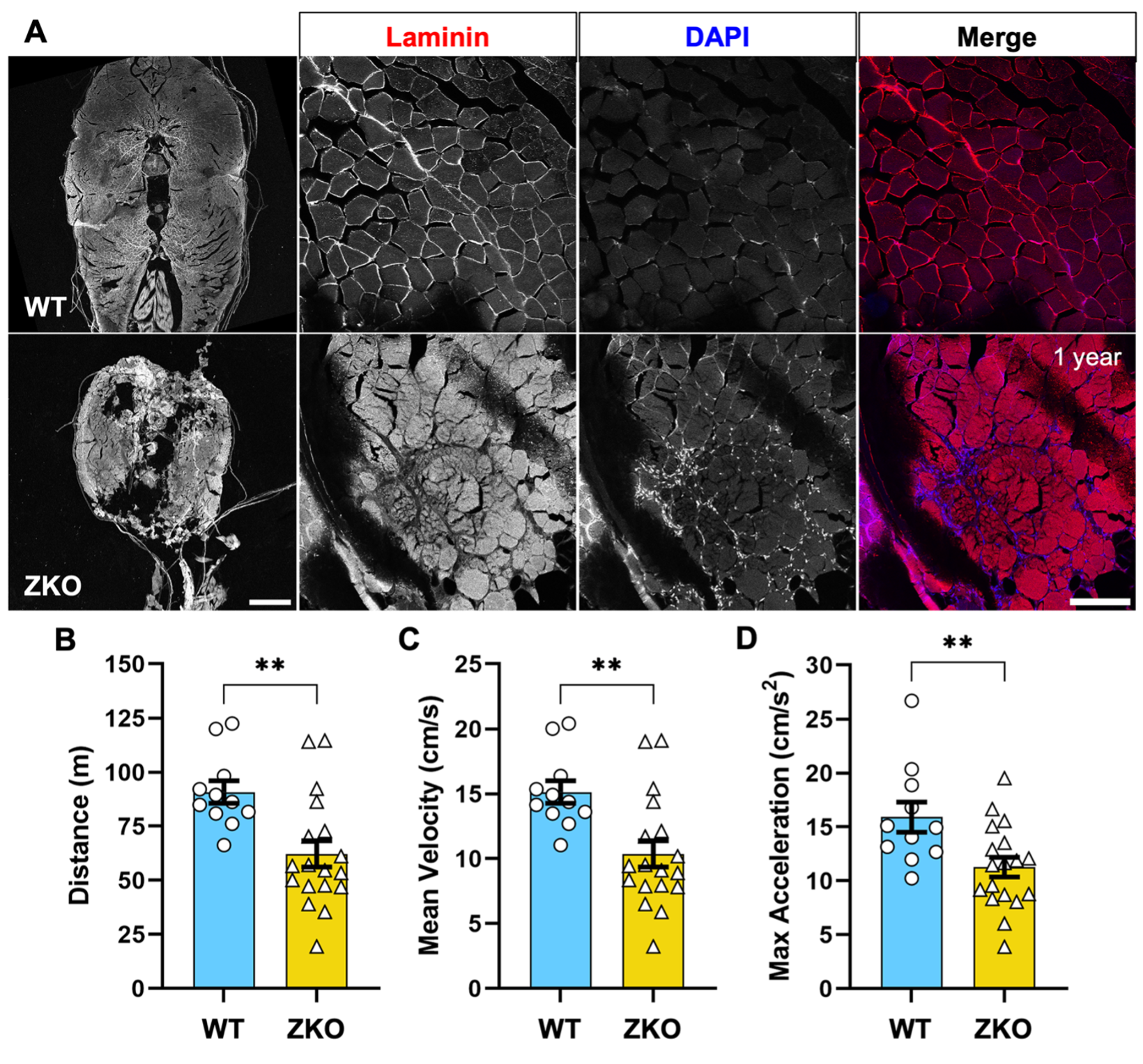

Supplement: S2 Fig — A: Full transverse cryosections showing complete deterioration of muscle integrity in ZKOs reflective of advanced muscle disease, in addition to disrupted laminin staining around myofibers and increased nuclear staining (DAPI) suggestive of severe fibrosis (Scale Bars: 500 µm for whole cryosection, 20 µm for zoomed-in images of muscle). B–D: Comprehensive analysis of swimming behavior and locomotor function showing that ZKOs have reductions in standard measures such as distance (B), velocity (C), and acceleration (D). (TIF) [file pgen.1011987.s002.tif]

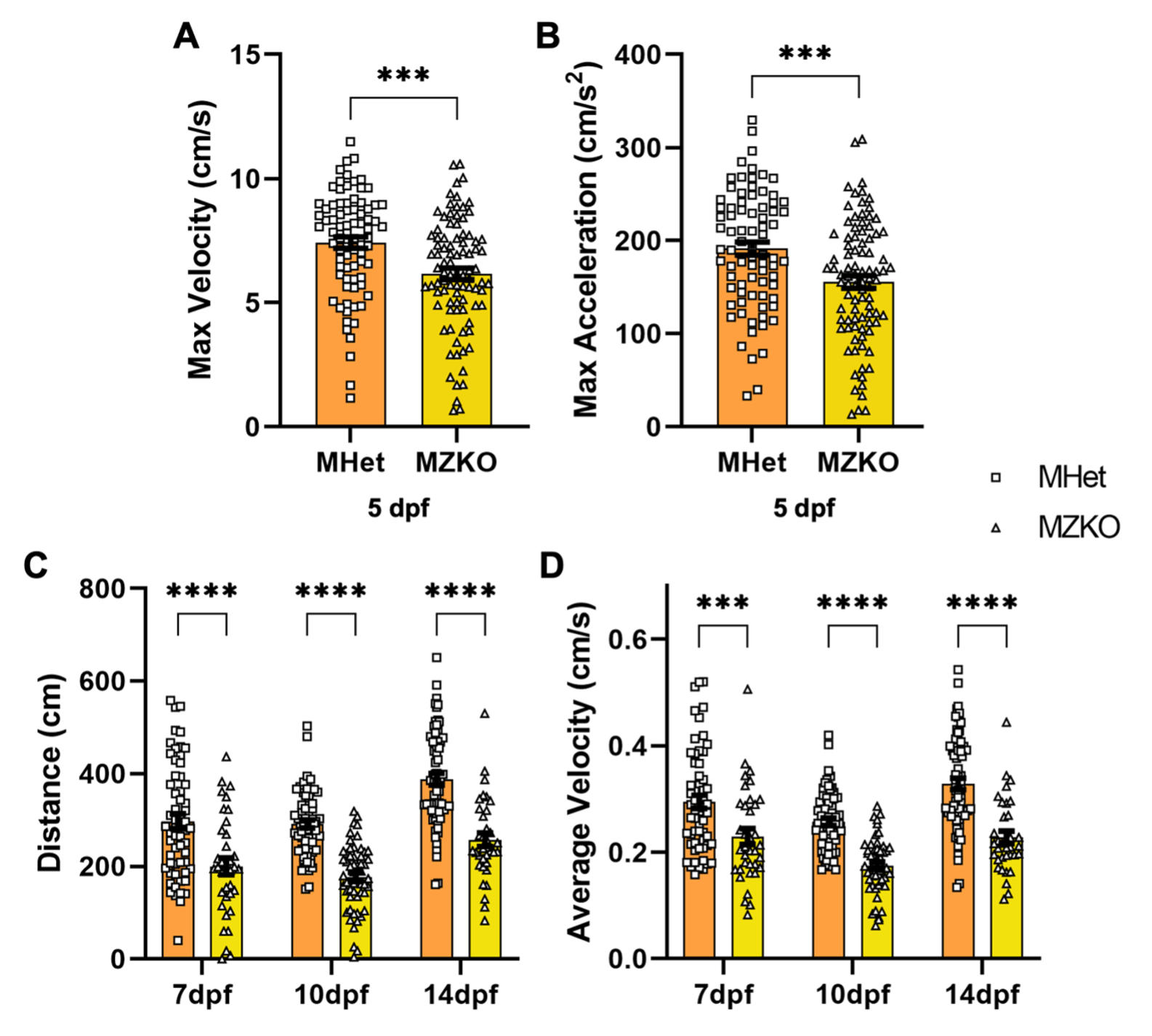

Supplement: S3 Fig — A, B: Assessment of maximum velocity (A) and maximum acceleration (B) at 5 dpf showing significant reductions in the MZKOs. C, D: Assessment of total distance (C) and average velocity (D) showing significant reductions in the MZKOs at 7, 10, and 14 dpf. (TIF) [file pgen.1011987.s003.tif]

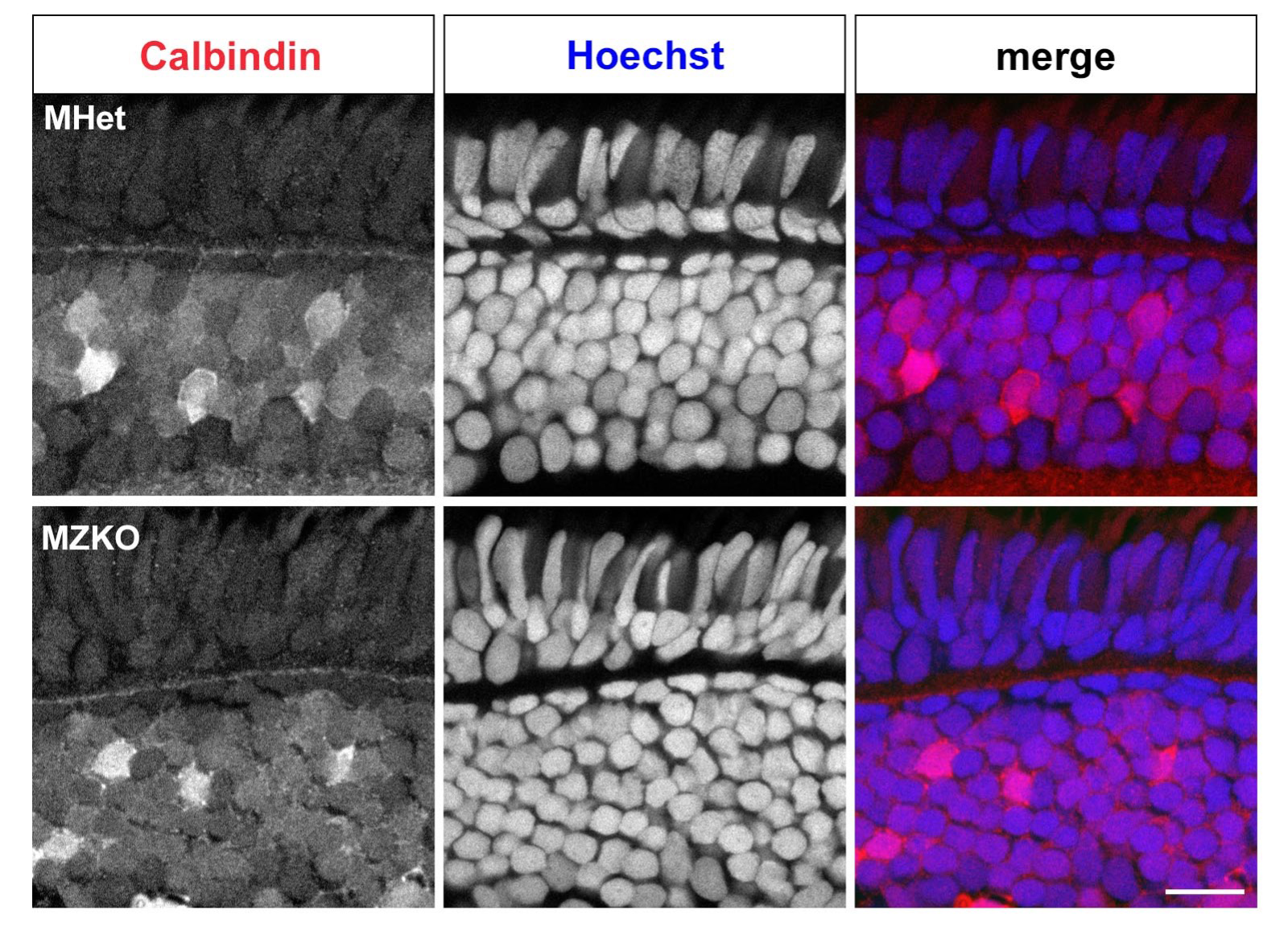

Supplement: S4 Fig — Retina sections from 10 dpf pomgnt2 MHet and MZKO fish showed no difference in calbindin immunostaining outlining horizontal cell processes and a subset of bipolar cells. (63X magnification, scale bar: 10μm). (TIF) [file pgen.1011987.s004.tif]

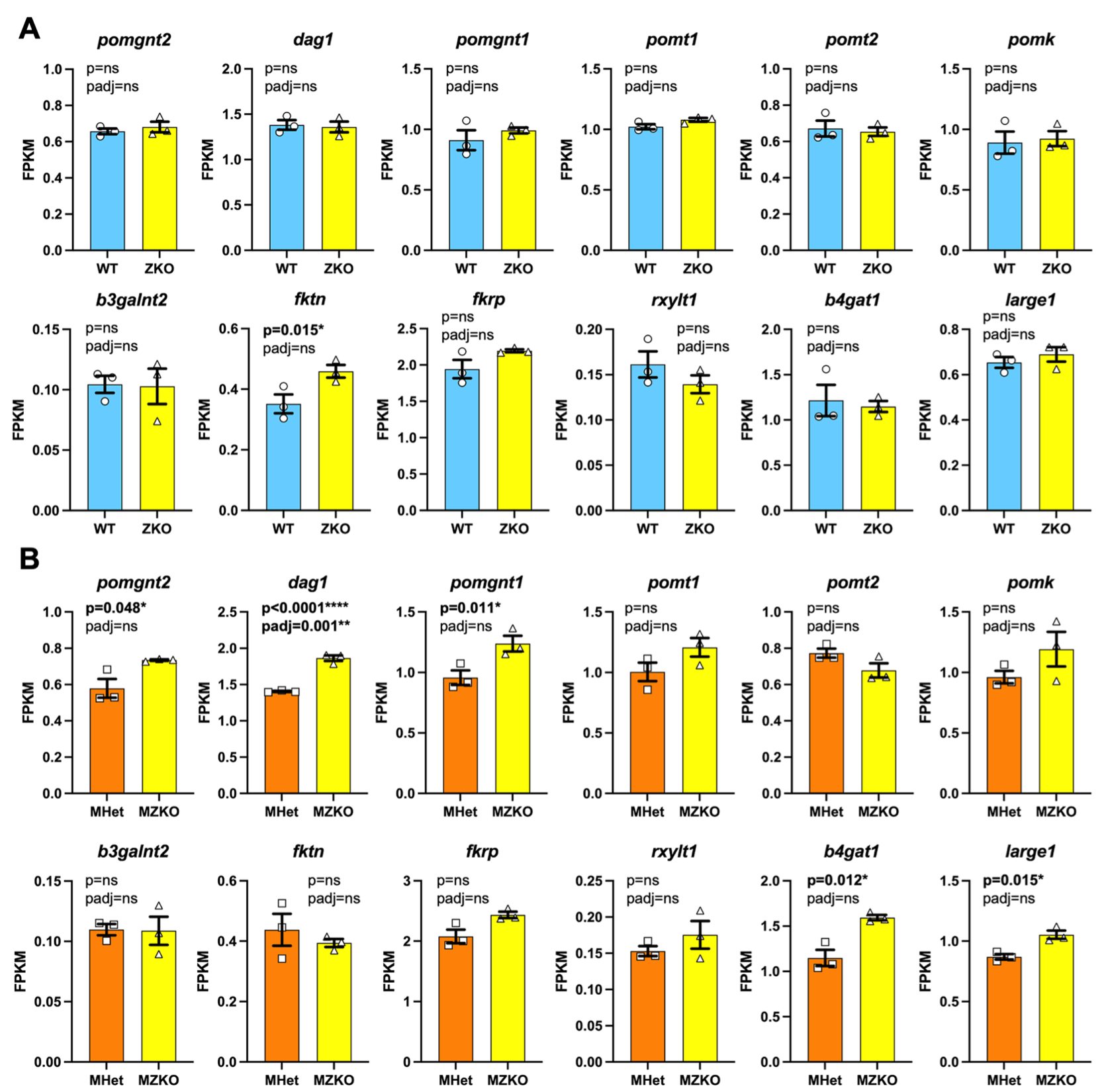

Supplement: S5 Fig — A, B: FPKM values with unadjusted and adjusted p-values derived from RNA-seq experiments from HetxHet crosses (A) and KOxHet crosses (B). (TIF) [file pgen.1011987.s005.tif]

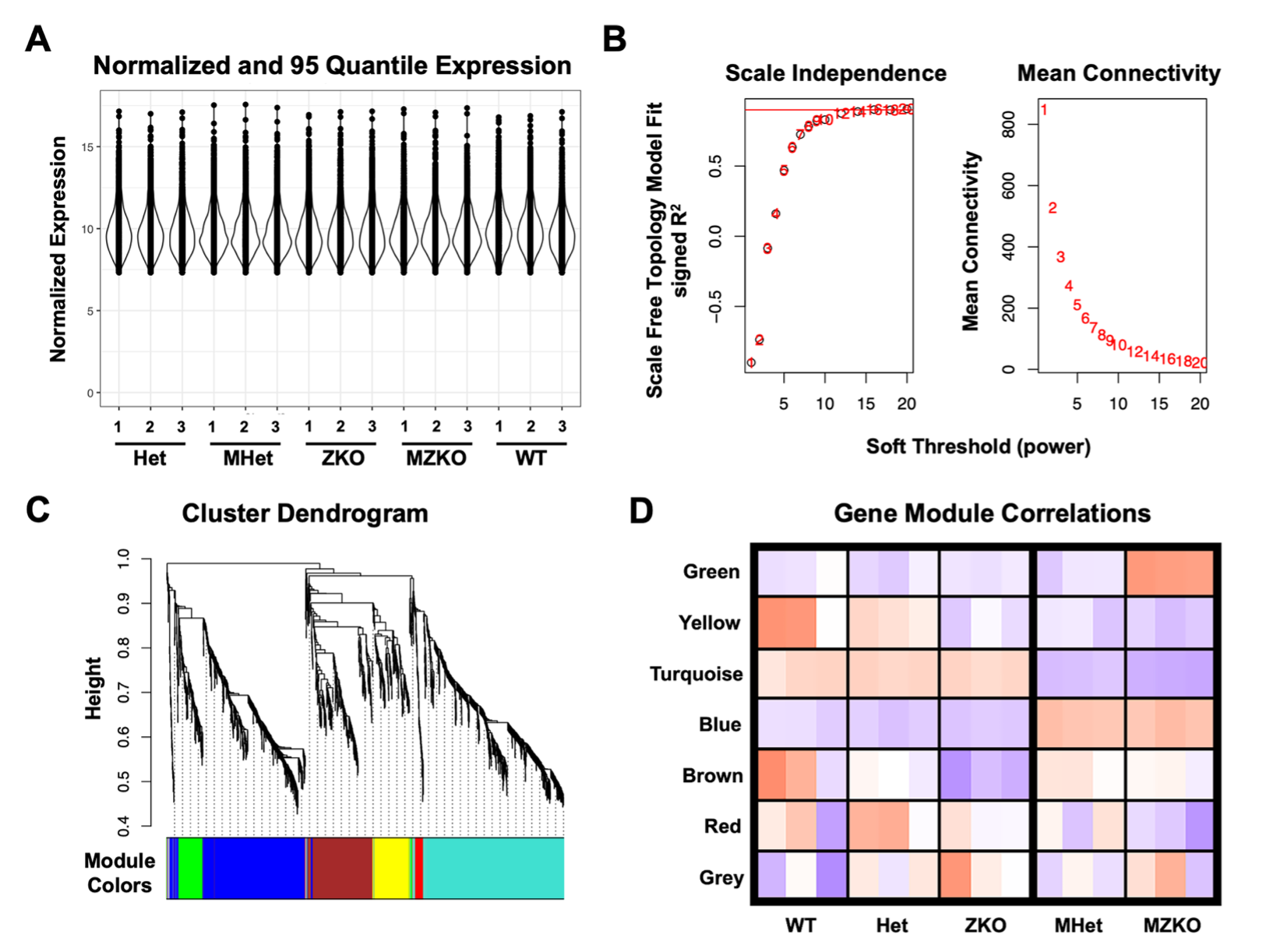

Supplement: S6 Fig — A: Data set normalization through DESeq2 and reduction to include genes expressed in the 95th quantile and above in all samples. B: Selection of soft thresholding power based on scale independence and mean connectivity. A soft thresholding value of 12 was selected. C: Dendrogram of gene modules derived from the reduced dataset of 1818 genes. The largest gene modules are turquoise and blue. D: Heat map of all gene modules identified, including those strongly correlated with maternal of zygotic genotype (green, yellow, turquoise, blue), and those that are not (brown, red, grey). (TIF) [file pgen.1011987.s006.tif]

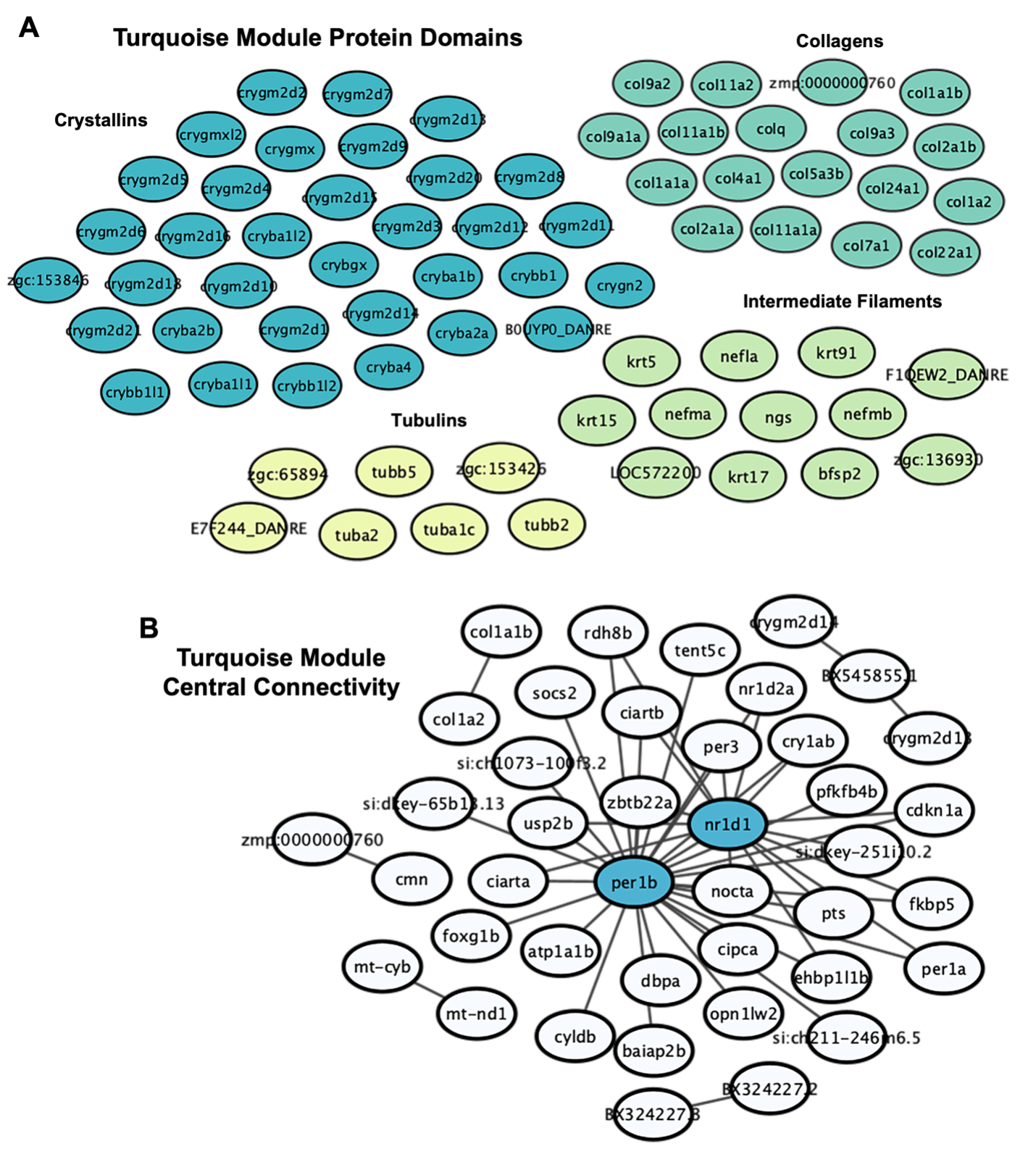

Supplement: S7 Fig — A: Enrichment of crystallin, collagen, tubulin, and intermediate filament protein domains. B: Expression correlation analysis of the turquoise module revealing per1b and nr1d1 to be the most centrally connected hub genes. (TIF) [file pgen.1011987.s007.tif]

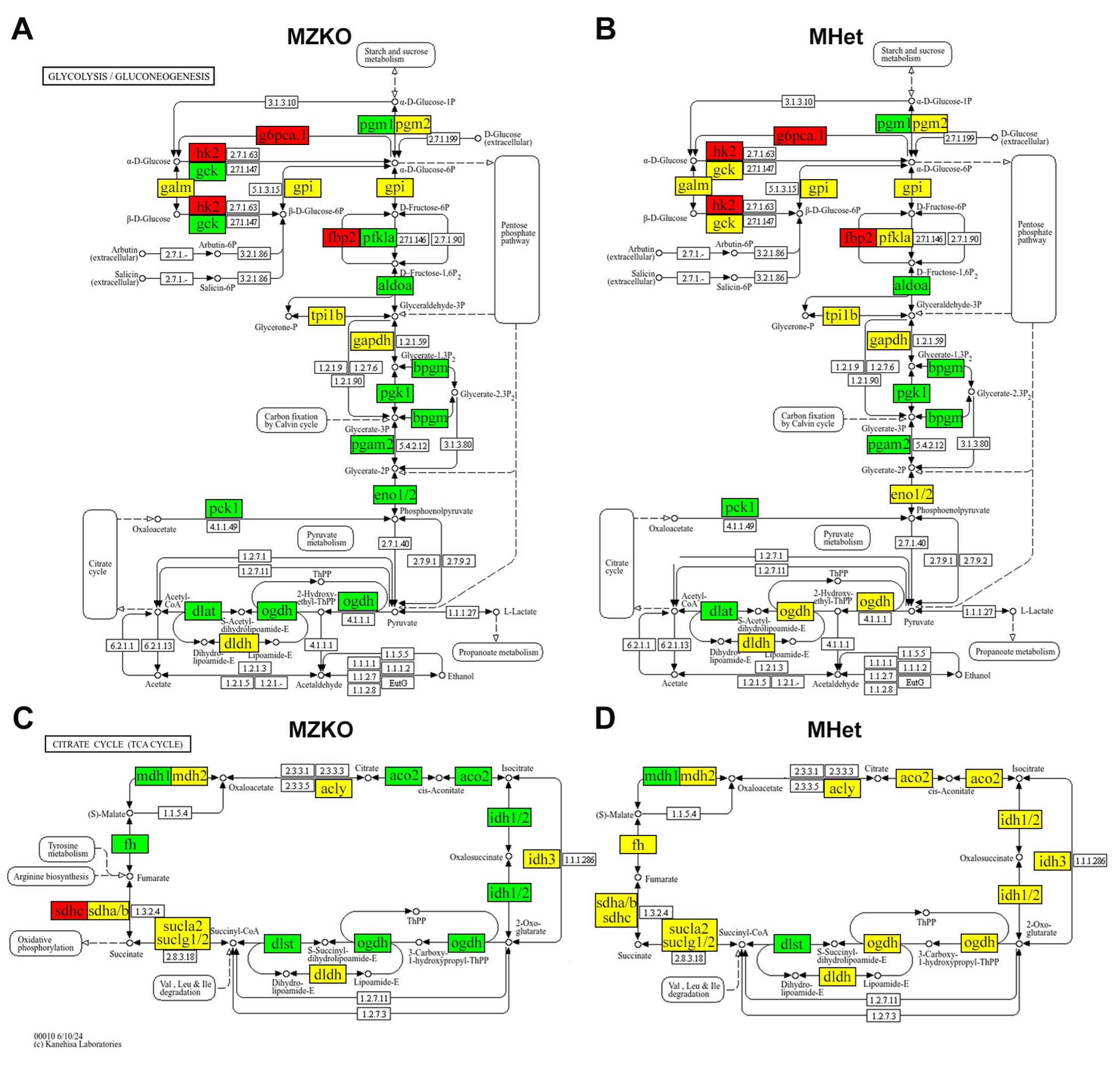

Supplement: S8 Fig — Transcripts tested in the arrays were mapped using KEGG Mapper (https://www.genome.jp/kegg/mapper/color.html) with respective KEGG identifiers and labeled in yellow when unchanged, red when increased and green when decreased. Outputs were modified by adding gene identifiers to ease identification. A. Glycolysis was overall reduced in MZKO with an increase in transcripts involved in gluconeogenesis. B. Similar changes were present in MHet with notable differences in gck and enolases leading to the TCA cycle. C, D. While the TCA cycle was affected in MZKO (C.), fewer changes were observed in MHet (D.). (TIF) [file pgen.1011987.s008.tif]
