# Supplementary material for: Impact of maternal compensation on developmental phenotypes in a zebrafish model of severe congenital muscular dystrophy
Source: PLoS Genet. 2026 Jan 14;22(1):e1011987. doi: 10.1371/journal.pgen.1011987 (PMC12803454; doi:10.1371/journal.pgen.1011987)
Supplement: Attachment — Submitted filename: FlanneryEtAl Response.docx [file pgen.1011987.s014.docx]

We thank the reviewers for their excitement about our study and for their thoughtful comments that have significantly improved our manuscript. Individual comments are addressed in blue below.

Reviewer #1: The manuscript by Flannery et al., centers on the generation and characterization of a zebrafish model of severe congenital muscular dystrophy by targeting protein O-mannose N-Acetylglucosaminyltransferase 2 (pomgnt2). POMGNT2 is a maternally provided gene that maintains cell-extracellular matrix interactions through glycosylation and leads to congenital muscular dystrophy (CMD) in human patients with pathogenic variants. The authors determined that zygotic knockouts (ZKOs) retain protein function in the first week post fertilization and survive to adulthood, though they develop muscle disease later in life. Interestingly, maternal-zygotic KOs (MZKOs) generated from ZKO females develop early-onset muscle disease, reduced motor function, neuronal axon guidance deficits, and retinal synapse disruptions recapitulating features of the human presentation. The offspring from a ZKO mother, independently of genotype, show distinct expression patterns from animals obtained from heterozygous breedings. The authors determined that these findings implicate genetic compensation and implicate maternal contributions in modeling disease using maternal-zygotic mutants.

This is a very interesting paper with implication for modeling congenital disorders of glycosylation (CDGs) and other human disorders with maternal mRNA contributions. There have been several analyses of previous zebrafish mutant strains that have demonstrated maternal contributions with differential phenotypes, but this is the first for a characterized CDG mutant. Some proper contextualization with other well-documented maternal-zygotic zebrafish mutants with differential phenotypes needs to be discussed. The experiments are logical with mostly appropriate controls and statistical analysis. This manuscript has the potential to be impactful in the CDG and maternal-zygotic impact of generational contributions of key mRNAs/genes.

General Comments:

1. It would be helpful to know if there is genetic compensation from other POMGNT2 pathway members, particularly POMK, POMT1/2, and POMGNT1.

We agree that this is valuable information for the reader to know in a model that is so well equipped for genetic compensation. We have examined expression of these genes through our transcriptomic analyses at 5 dpf in ZKOs and at 5 and 10 dpf in MZKOs (new Supplemental Figure 5), and we did not find any evidence to suggest that they were genetically compensating for loss of *pomgnt2*. We have added mention of these findings in the Results section.

2. The proposed increased energy demand on the ZKO pomgnt2 mutants is an interesting hypothesis, but do the authors actually have data showing increased metabolic demand via ATP measurement comparisons or NAD+ level changes?

Since changed in metabolic genes were in multiple directions, we decided to perform a more comprehensive assessment of 84 genes involved in glucose metabolism using Qiagen RT2 Profiler arrays. This approach revealed metabolic reprogramming in the MZKO leading to a reduction in glycolysis and TCA cycle with an increase in gluconeogenesis and of several genes in the pentose phosphate pathway. Some of these changes were also present in the MHet but at a lesser extent. In addition, since the inflammatory marker beta-2-microglobulin was included in the control, we found increases in both the MZKO and MHet. We realized that the increase in energy demand conclusion purely based on WGCNA was premature, but we revealed that MHet have baseline change that may be primed for metabolic reprogramming and inflammation. We have rewritten the Results section and Discussion to reflect this new finding.

3. Have the authors quantitatively evaluated the levels of pomgnt2 mRNA in the maternal KO mutants? Essentially is there any lingering mRNA from the mother that may account for differential phenotypes from the ZKO mutants?

*pomgnt2* mRNA levels were examined in ZKOs at 5 dpf through RNA-seq, and at 24, 48, 72 and 96 hpf through qPCR (new Fig. 2A). Expression was also examined in MZKOs at 5 and 10 dpf through RNA-seq. In each experiment, the evidence shows that *pomgnt2* does not undergo nonsense mediated decay despite the frameshift mutations rendering the Pomgnt2 protein nonfunctional, which we see through staining with IIH6C4 for matriglycan. We reexamined the reads from the RNAseq at 5 and 10 dpf and only mutated mRNA is present.

4. Can the authors elaborate a bit more on any neuronal/brain phenotypes, e.g. cobblestone lissencephaly that often occurs in glycosylation disorders.

Overall, brain phenotypes in zebrafish seem to be limited to conserved axon guidance pathways. In the Discussion, we have now elaborated more on the *Pomgnt2* KO mouse model which develops cortical malformations reflective of cobblestone lissencephaly and on the evolutionary differences in brain development between fish and humans, including the absence of a cerebral cortex, that could lead to these phenotypic differences.

5. There’s a large amount of literature on differential phenotypes in zebrafish mutant lines due to maternal-zygote effects. Some famous examples are MZzoep, MZspg, mater, and many other early pluripotency genes. The authors should really discuss these cases more and put them in proper contextualization in the discussion section.

This is an important consideration as *pomt1* and *pomgnt2* appear to belong to a more long-acting class of transcripts than those establishing zygotic genome activation and early embryonic patterning. We added a paragraph in the Discussion touching on some of these differences and possible mechanisms.

6. I’m also a bit surprised that the authors did not compare and contrast more in depth the corresponding authors’ previous work on the pomgnt2/gtdc2 morphant zebrafish (Manzini et al., AJHG, 2012). Given the impact of mRNA contributions, the authors should discuss the phenotypes in a more detailed comparative analysis.

This is a great suggestion and opens up a very interesting discussion on morphant and mutant phenotypes, even when the maternal deposit is accounted for. We have added this information to the Discussion, where we mention how *pomgnt2* morphants show severe developmental defects by 3 dpf, while *pomgnt2* MZKOs show severe disease phenotypes between 1-2 weeks.

Reviewer #2: In this study, the authors generate and characterize novel zebrafish mutants of pomgnt2, a gene required for the glycosylation of Dystroglycan. Defects in Dystroglycan glycosylation lead to a form of congenital muscular dystrophy, and zebrafish mutants recapitulate both the muscle and CNS pathology seen in human patients and rodent models, making them a good genetic model for the disease. Previous work from this group had found that maternal deposition of mRNA can compensate for loss of pomt1 (another gene required for Dystroglycan glycosylation) in zygotic knockouts. Here they show that this is also the case for pomgnt2: zygotic mutants have a very mild phenotype, whereas mutants generate from KO mothers (maternal-zygotic KOs) show more severe phenotypes that manifests as early as 5 days post-fertilization. They provide a nice side-by-side comparison of these zygotic and maternal-zygotic knockouts, and conduct transcriptomic analysis that identifies a large number of dysregulated genes/pathways in both models that appear before behavioral and anatomical phenotypes are apparent. Overall, this study is well-designed and straightforward, the data are clear, and the findings provide important considerations for how zebrafish models of dystroglycanopathy can be used. There are a couple of issue that should be addressed prior to publication:
• Figure 1: the authors should provide a schematic showing how the crispr frameshift mutations would affect the predicted amino acid sequence

Done. We have now added the predicted frameshift amino acid sequence to Figure 1A that is predicted from the frameshift mutations.

• Figure 1 shows immunostaining for matriglycan (IIH6 antibody) and DAPI. This data nicely shows the loss of glycosylated Dystroglycan in the mutants, but the "variation in myofiber size, gaps between myofibers" (line 135) cannot really be seen here. Can the authors provide a different type of stain (perhaps H&E or laminin) to show the myofibers? Also, they state "increased nuclear staining suggesting fibrosis" (line 136). The increased nuclear staining needs to be quantified.

We appreciate the thoughtful critique. In addition to IIH6C4 staining shown in Fig 1E, we have now added two additional panels showing phalloidin (Fig 1F) and laminin (Fig 1G) staining which recapitulate the fiber disorganization and variability that we previously observed. With each of these stains, we have also provided additional nuclear staining with either DAPI or Hoechst and have modified our language to state that we observe “nuclear staining patterns suggestive of fibrosis” rather than “increased nuclear staining”.

• Re: Westerns in Fig 1 and S1, the WT samples have significantly less B-DG in all of the westerns provided. Is this a phenotype, or just due to unequal loading? Is there a loading control for these westerns?

The western blots presented were performed through wheat germ agglutinin enrichment of glycoproteins and β-DG is very commonly used as a loading control to IIH6C4 detection as it gets enriched among the glycoproteins in the lysate. We initially interpreted the increased β-DG detection as an unequal loading issue that actually made the loss of IIH6C4 even more significant, but re-examination of our total protein stains (amido black) indicate that this could actually be an increase in β-DG expression. In reanalyzing gene expression following a comment from Rev 1, we have found a significant increase in *dag1* which could explain the increased enrichment in Fig. 1B noted by this reviewer and have added this information in the text and in new Supplemental Fig. 5.

• There were a couple points (line 172, 184) where a "trend" towards a difference between genotypes was reported at 10 and 14dpf. These differences are very small and not statistically significant (as opposed to 28dpf). Please remove all mentions of "trends towards significance".

Done. We have removed any mention of a “trend” toward significance and have instead stated that the reductions in body length at 10 and 14 dpf were not statistically significant.

• Interesting that IIH6 is completely gone in the MZKO fish at P7 (Figure 2A), but laminin staining in the muscle still looks fairly normal at this time point (Figure 3E). Could the author comment on this in the discussion? Perhaps there are some clues to upregulated pathways in their transcriptomic analysis that provide clues to how laminin is initially organized in the absence of glycosylated Dystroglycan.

Dystroglycan is not necessary for assembly of the myotendinous junctions (MTJ) and multiple mutants show normal initial assembly with later loss of muscle fiber attachment. This is described in the Discussion, especially in relation to the *lama2* mutants where the MTJ is present, but fibers begin to detach a few days post fertilization.

• The quantification in Figure 4 shows that the BTX:SV2 colocalization is more severely disrupted at the MTJ compared to the myofibers. I suggest adding a higher magnification inset for these, as this is somewhat hard to distinguish at the current magnification.

Done. We have now provided a higher magnification inset in Fig. 4 that more clearly demonstrates this finding.

• The authors state: "Horizontal cell nuclei lining the upper border of the inner nuclear layer (INL) also appeared more diffuse and disorganized in MZKOs (Fig. 5A)." (Line 273-275). How do they know these are horizontal cells, as the current staining is only for DAPI? Could they verify this with a horizontal cell marker?

We have analyzed horizontal cell processes using calbindin staining in additional mutants as we had done in pomt1 MZKOs and we found that they were preserved (new Suppl. Fig. 4). We have revised our statement in light of this data.

Minor points:
• Supplemental Figure 4 and Figure 8 are intimately related to one another. For ease of readership, it makes sense to name the modules referenced in lines 376-377 by name (ie. blue, green, etc.) so that it is less jarring when the green module is mentioned in the following paragraph if the reader has not downloaded/referenced the supplements.

We agree with the reviewer, and in order to make these results as fluid and easy to follow as possible for the readers, we decided to move the graphs outlining the fold changes for genes in each model (originally Supplemental Fig 4E-H) into the main figure (now Fig. 8B-E). This way, the correlations shown within the heat map are referenced, followed by each graph clearly depicting the expression changes. We believe this will enhance the reader’s understanding of the complex correlations that follow.

• Please double check gene name capitalization conventions throughout the introduction and discussion sections. If referring to a human or mouse gene name, capitalize the first letter. If referring to a human gene symbol, capitalize every letter. If referring to a mouse gene symbol, capitalize only the first letter.

We apologize for the confusion. Gene name capitalization conventions have now been updated in accordance with species-specific conventions.

• Line 61 refers to Dystroglycan as the extracellular component of the Dystrophin Glycoprotein Complex; it would be more appropriate to refer to Dystroglycan as the transmembrane component of the complex

We have revised this description to indicate that α-DG is extracellular while β-DG is the transmembrane subunit encoded by the same protein.

• The manuscript could benefit from some closer copy editing. There were typos and errors throughout the discussion and methods sections. In the main text the Figure 6 figure legend erroneously refers to panels D-E. They should be C-D.

This specific error has been corrected, and we have now carefully copyedited the manuscript to address additional typos and errors.

Some questions regarding the methods:
o. What objectives were used for image acquisition (magnification, NA, air vs immersion)?

Objectives for image acquisition are now described in the methods section for the images and details on magnification have also been added to each respective figure legend where an image is included.

o Within the “Weighted gene co-expression network analysis section” it is stated that RStudio was used to perform analysis. RStudio is just the IDE, what version of R was used?

R version 4.4.1 was used. This is now updated in this section of the methods, as well as the RNA sequencing and analysis section where R is referred to without a version.

o In the “Quantitative analysis of neuromuscular junctions” section the image acquisition parameters are described including the following sentence: “For each slice, the sample area was scanned four times and averaged with 1 μm intervals.” What does the “averaged with 1um intervals” refer to? Is this pixel binning? Or z-plane averaging? Or does the 1um simply refer to the interval between z-planes, unrelated to the averaging? Please clarify.

This wording is now clarified. Z-stacks were obtained with 1 um intervals between planes, while each plane was scanned four times and averaged.

o The “Statistical analysis” section mentions the following: “For comparisons between 2 groups of a single measure, an unpaired t-test was used, or alternatively a Mann-Whitney test for nonparametric datasets.” Datasets are not described as nonparametric. Datasets are non-normal. Statistical tests are nonparametric.

This wording has now been corrected.

o Also in the “Statistical analysis” section - is there a reference for the ROUT method?

The Robust Regression and Outlier Removal test, also known as the ROUT method, is recommended by GraphPad for outlier identification. The methodology paper outlining this method (Motulsky & Brown, 2006) has now been cited in the text.
